# Supplementary material for: De Novo Transcriptome Assembly of Two Microsorum Fern Species Identifies Enzymes Required for Two Upstream Pathways of Phytoecdysteroids
Source: Int J Mol Sci. 2021 Feb 19;22(4):2085. doi: 10.3390/ijms22042085 (PMC7923240; doi:10.3390/ijms22042085)

Supplementary

De Novo Transcriptome Assembly of Two *Microsorum* Fern Species Identifies Enzymes Required for Two Upstream Pathways of Phytoecdysteroids

Siriporn Sripinyowanich [1], Eui-Joon Kil [2], Sahanat Petchsri [1], Yeonhwa Jo [3], Hoseong Choi [3], Won Kyong Cho [3,*,†] and Sukchan Lee [4,*,†]

[1] Department of Botany, Faculty of Liberal Arts and Science, Kasetsart University, KamphaengSaen Campus, NakhonPathom 73140, Thailand; siriporn.srip@ku.th (S.S.); faassnps@ku.ac.th (S.P.)
[2] Department of Plant Medicals, Andong National University, Andong 36729, Korea; viruskil@anu.ac.kr
[3] Research Institute of Agriculture and Life Sciences, College of Agriculture and Life Sciences, Seoul National University, Seoul 08826, Korea; yeonhwajo@gmail.com (Y.J.); bioplanths@snu.ac.kr (H.C.); wonkyong@gmail.com (W.K.C.)
[4] Department of Integrative Biotechnology, Sungkyunkwan University, Suwon 16419, Korea; cell4u@skku.edu (S.L.)
* Correspondence: wonkyong@gmail.com (W.K.C.); cell4u@skku.edu (S.L.)
† These authors contributed equally to this work.

Supplementary data

Supplementary Tables

Table S1. Results of differential gene expression analysis using DESeq2 program.
Table S2. The list of differentially expressed transcription factor genes between MP and MS.
Table S3. Protein sequences involved in PEs biosynthesis identified from two *Microsorum* transcriptomes.
Table S4. Transcript abundance for identified transcripts in MP.
Table S5. Transcript abundance for identified transcripts in MS.
Table S6. Primer sequences used for real time RT-PCR.
Table S7. The information of 31 drosophila CYP proteins.
Table S8. Protein sequences of identified CYP proteins from MP.
Table S9. Protein sequences of identified CYP proteins from MS.

Supplementary Figures

Figure S1. Experimental scheme for construction of two *Microsorum* reference transcriptomes. (a) Fronds from two different *Microsorum* species, *M. punctatum* (MP) and *M. scolopendria* (MS) were used for the preparation of the mRNA library. (b) HPLC analysis was conducted to measure phytoecdysteroids (PEs). (c) Paired-end sequenced reads by NovaSeq 6000 system were subjected to de novo transcriptome assembly followed by the deletion of contaminated sequences. (d) Clean transcripts associated with Microsorum were used for functional annotation. (e) Based on functional annotation and BLAST search, we identified putative enzymes in two *Microsorum* species involved in PEs biosynthesis.

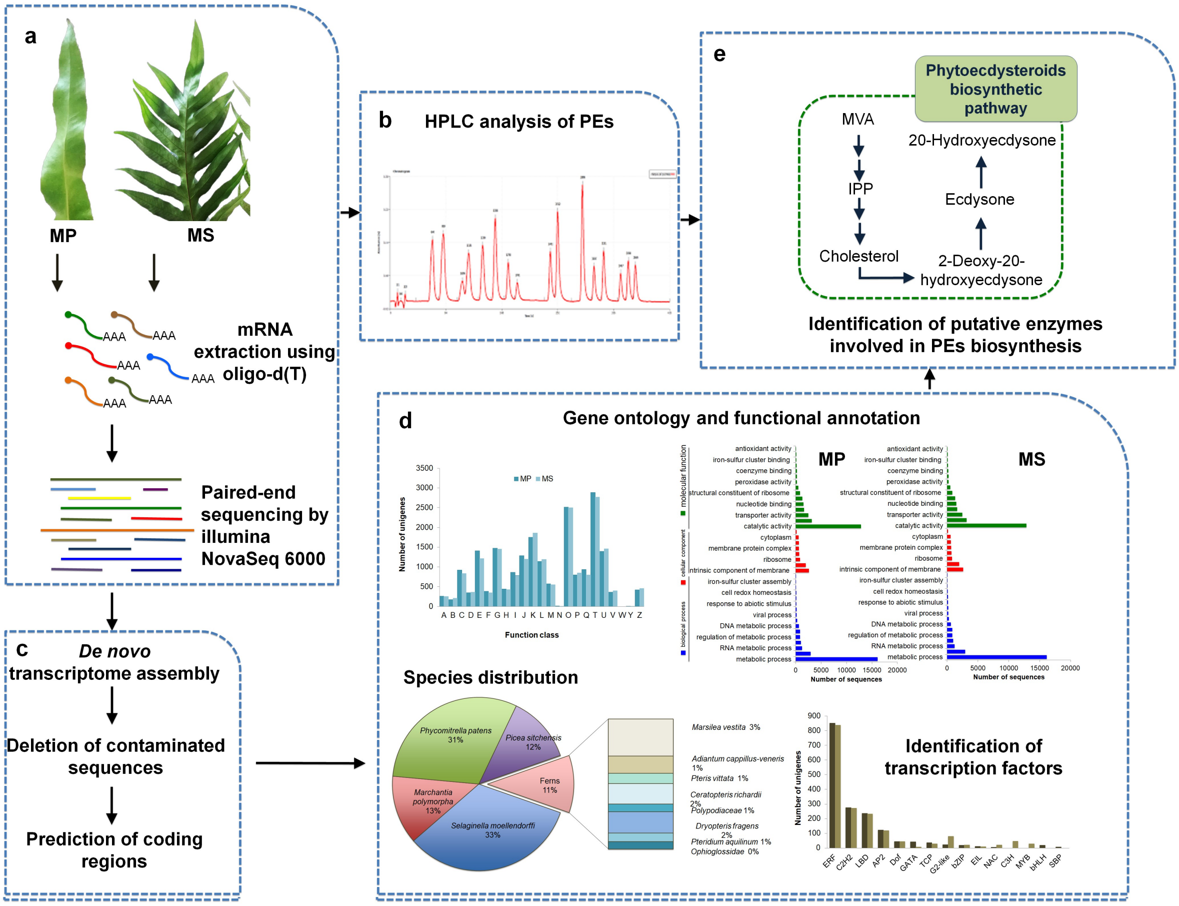

Figure S2. The relative expression levels of 11 transcripts encoding HMGS1, MVK, PMK, CAS, DHCR7, EBP, ERG-1, ERG-2, IDI, MVD, and SQS were measured by real-time RT-PCR. Red and blue bars indicate MP and MS, respectively. For the comparisons between MP and MS, we used independent T-tests. Asterisks represent significant differences (* $p < 0.05$, ** $p < 0.01$, and *** $p < 0.001$).

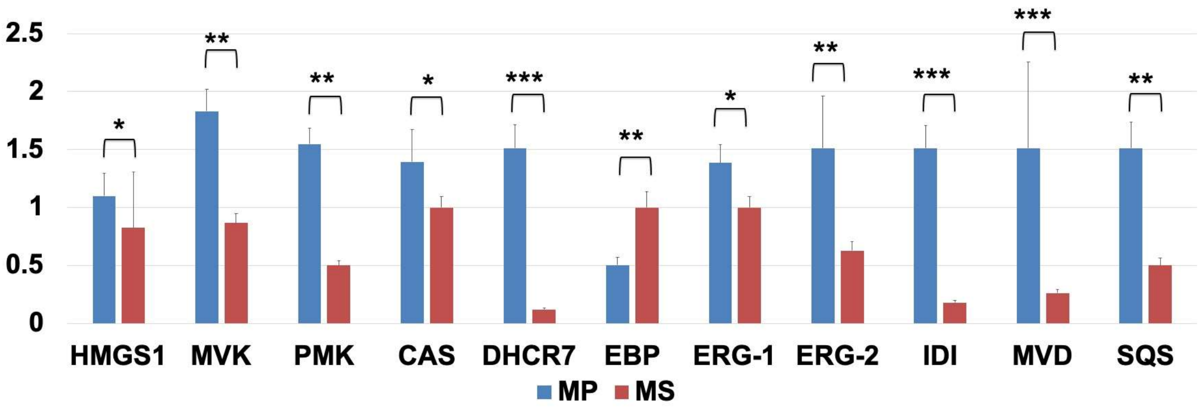

Figure S3. Phylogenetic tree of 146 CYP proteins identified from MP with the 31 drosophila CYP proteins. Protein sequences were aligned using MAFFT. The phylogenetic tree was generated by IQ-TREE using maximum likelihood method, LG+I+G4 substitution model, and bootstrap with 1,000 iterations. The phylogenetic tree was visualized by the FigTree (version 1.4.4) program.

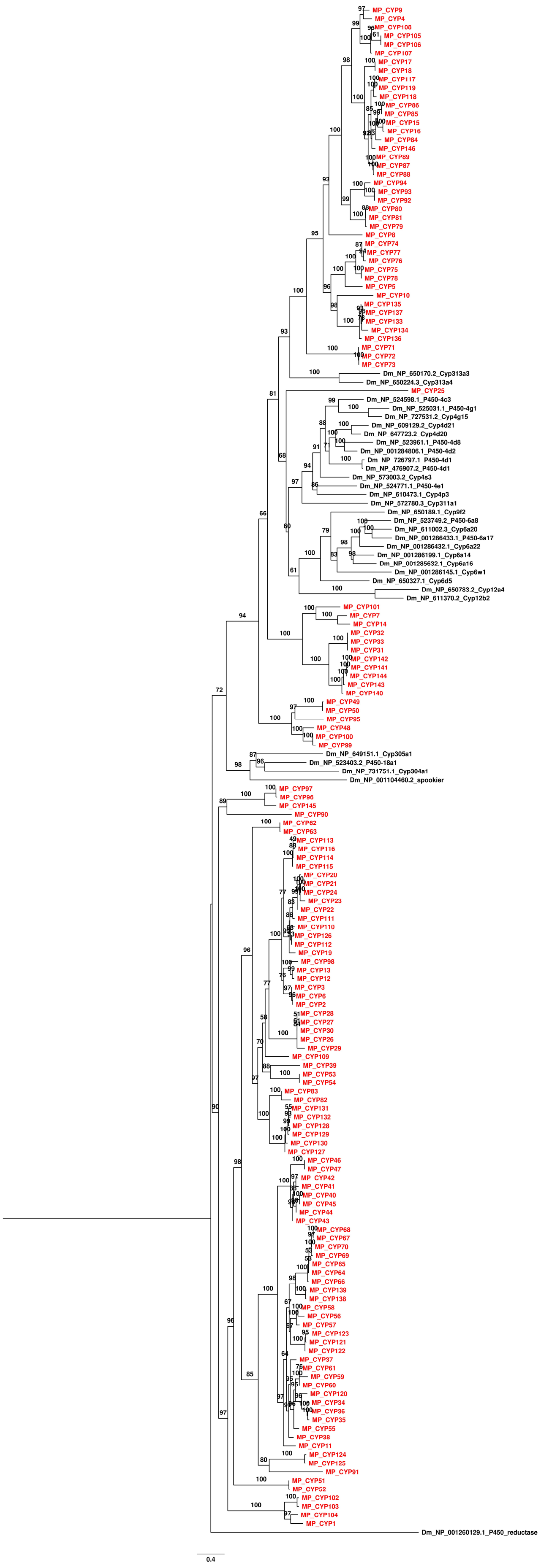
MP_CYP9
MP_CYP4
MP_CYP108
MP_CYP105
MP_CYP106
MP_CYP107
MP_CYP17
MP_CYP18
MP_CYP117
MP_CYP119
MP_CYP118
MP_CYP86
MP_CYP85
MP_CYP15
MP_CYP16
MP_CYP84
MP_CYP146
MP_CYP89
MP_CYP87
MP_CYP88
MP_CYP94
MP_CYP93
MP_CYP92
MP_CYP80
MP_CYP81
MP_CYP79
MP_CYP8
MP_CYP74
MP_CYP77
MP_CYP76
MP_CYP75
MP_CYP78
MP_CYP5
MP_CYP10
MP_CYP135
MP_CYP137
MP_CYP133
MP_CYP134
MP_CYP136
MP_CYP71
MP_CYP72
MP_CYP73
Dm_NP_650170.2_Cyp313a3
Dm_NP_650224.3_Cyp313a4
MP_CYP25
Dm_NP_524598.1_P450-4c3
Dm_NP_525031.1_P450-4g1
Dm_NP_727531.2_Cyp4g15
Dm_NP_609129.2_Cyp4d21
Dm_NP_647723.2_Cyp4d20
Dm_NP_523961.1_P450-4d8
Dm_NP_001284806.1_P450-4d2
Dm_NP_726797.1_P450-4d1
Dm_NP_476907.2_P450-4d1
Dm_NP_573003.2_Cyp4s3
Dm_NP_524771.1_P450-4e1
Dm_NP_610473.1_Cyp4p3
Dm_NP_572780.3_Cyp311a1
Dm_NP_650189.1_Cyp9f2
Dm_NP_523749.2_P450-6a8
Dm_NP_611002.3_Cyp6a20
Dm_NP_001286433.1_P450-6a17
Dm_NP_001286432.1_Cyp6a22
Dm_NP_001286199.1_Cyp6a14
Dm_NP_001285632.1_Cyp6a16
Dm_NP_001286145.1_Cyp6w1
Dm_NP_650327.1_Cyp6d5
Dm_NP_650783.2_Cyp12a4
Dm_NP_611370.2_Cyp12b2
MP_CYP101
MP_CYP7
MP_CYP14
MP_CYP32
MP_CYP33
MP_CYP31
MP_CYP142
MP_CYP141
MP_CYP144
MP_CYP143
MP_CYP140
MP_CYP49
MP_CYP50
MP_CYP95
MP_CYP48
MP_CYP100
MP_CYP99
Dm_NP_649151.1_Cyp305a1
Dm_NP_523403.2_P450-18a1
Dm_NP_731751.1_Cyp304a1
Dm_NP_001104460.2_spookier
MP_CYP97
MP_CYP96
MP_CYP145
MP_CYP90
MP_CYP62
MP_CYP63
MP_CYP113
MP_CYP116
MP_CYP114
MP_CYP115
MP_CYP20
MP_CYP21
MP_CYP24
MP_CYP23
MP_CYP22
MP_CYP111
MP_CYP110
MP_CYP126
MP_CYP112
MP_CYP19
MP_CYP98
MP_CYP13
MP_CYP12
MP_CYP3
MP_CYP6
MP_CYP2
MP_CYP28
MP_CYP27
MP_CYP30
MP_CYP26
MP_CYP29
MP_CYP109
MP_CYP39
MP_CYP53
MP_CYP54
MP_CYP83
MP_CYP82
MP_CYP131
MP_CYP132
MP_CYP128
MP_CYP129
MP_CYP130
MP_CYP127
MP_CYP46
MP_CYP47
MP_CYP42
MP_CYP41
MP_CYP40
MP_CYP45
MP_CYP44
MP_CYP43
MP_CYP68
MP_CYP67
MP_CYP70
MP_CYP69
MP_CYP65
MP_CYP64
MP_CYP66
MP_CYP139
MP_CYP138
MP_CYP58
MP_CYP56
MP_CYP57
MP_CYP123
MP_CYP121
MP_CYP122
MP_CYP37
MP_CYP61
MP_CYP59
MP_CYP60
MP_CYP120
MP_CYP34
MP_CYP36
MP_CYP35
MP_CYP55
MP_CYP38
MP_CYP11
MP_CYP124
MP_CYP125
MP_CYP91
MP_CYP51
MP_CYP52
MP_CYP102
MP_CYP103
MP_CYP104
MP_CYP1
Dm_NP_001260129.1_P450_reductase
0.4

Figure S4. Phylogenetic tree of 141 CYP proteins identified from MS with the 31 drosophila CYP proteins. Protein sequences were aligned using MAFFT. The phylogenetic tree was generated by IQ-TREE using maximum likelihood method, LG+I+G4 substitution model, and bootstrap with 1,000 iterations. The phylogenetic tree was visualized by the FigTree (version 1.4.4) program.

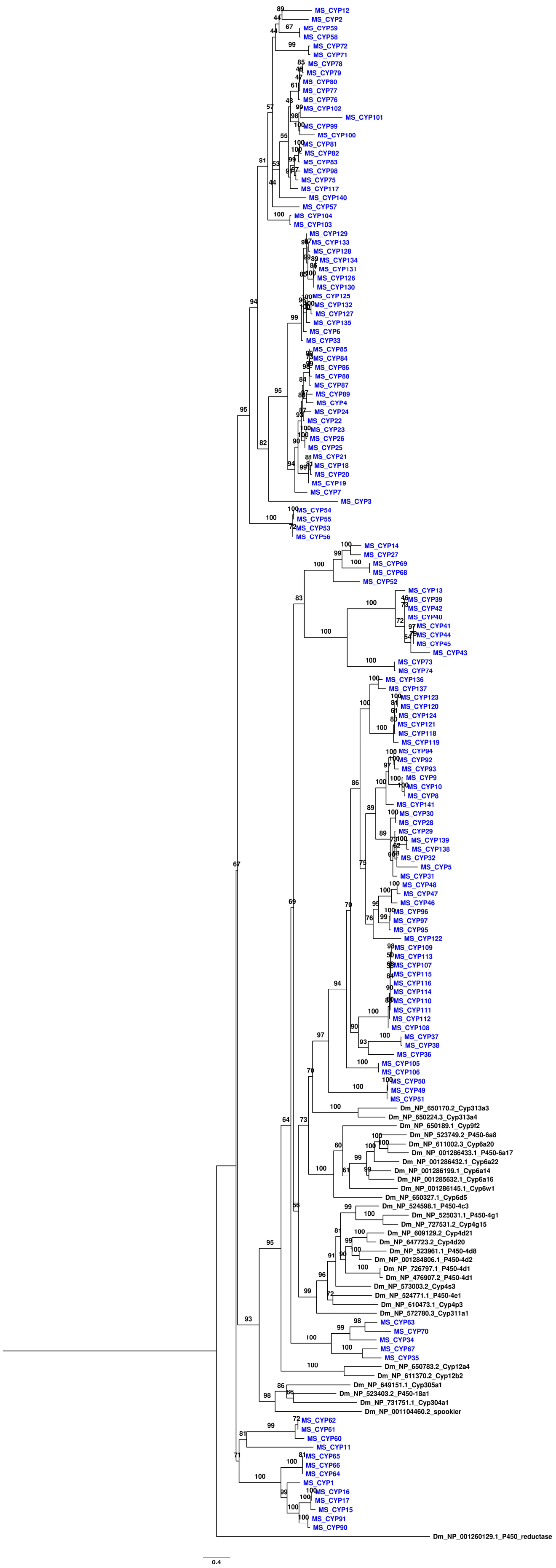
MS_CYP12
MS_CYP2
MS_CYP59
MS_CYP58
MS_CYP72
MS_CYP71
MS_CYP78
MS_CYP79
MS_CYP80
MS_CYP77
MS_CYP76
MS_CYP102
MS_CYP101
MS_CYP99
MS_CYP100
MS_CYP81
MS_CYP82
MS_CYP83
MS_CYP98
MS_CYP75
MS_CYP117
MS_CYP140
MS_CYP57
MS_CYP104
MS_CYP103
MS_CYP129
MS_CYP133
MS_CYP128
MS_CYP134
MS_CYP131
MS_CYP126
MS_CYP130
MS_CYP125
MS_CYP132
MS_CYP127
MS_CYP135
MS_CYP6
MS_CYP33
MS_CYP85
MS_CYP84
MS_CYP86
MS_CYP88
MS_CYP87
MS_CYP89
MS_CYP4
MS_CYP24
MS_CYP22
MS_CYP23
MS_CYP26
MS_CYP25
MS_CYP21
MS_CYP18
MS_CYP20
MS_CYP19
MS_CYP7
MS_CYP3
MS_CYP54
MS_CYP55
MS_CYP53
MS_CYP56
MS_CYP14
MS_CYP27
MS_CYP69
MS_CYP68
MS_CYP52
MS_CYP13
MS_CYP39
MS_CYP42
MS_CYP40
MS_CYP41
MS_CYP44
MS_CYP45
MS_CYP43
MS_CYP73
MS_CYP74
MS_CYP136
MS_CYP137
MS_CYP123
MS_CYP120
MS_CYP124
MS_CYP121
MS_CYP118
MS_CYP119
MS_CYP94
MS_CYP92
MS_CYP93
MS_CYP9
MS_CYP10
MS_CYP8
MS_CYP141
MS_CYP30
MS_CYP28
MS_CYP29
MS_CYP139
MS_CYP138
MS_CYP32
MS_CYP5
MS_CYP31
MS_CYP48
MS_CYP47
MS_CYP46
MS_CYP96
MS_CYP97
MS_CYP95
MS_CYP122
MS_CYP109
MS_CYP113
MS_CYP107
MS_CYP115
MS_CYP116
MS_CYP114
MS_CYP110
MS_CYP111
MS_CYP112
MS_CYP108
MS_CYP37
MS_CYP38
MS_CYP36
MS_CYP105
MS_CYP106
MS_CYP50
MS_CYP49
MS_CYP51
Dm_NP_650170.2_Cyp313a3
Dm_NP_650224.3_Cyp313a4
Dm_NP_650189.1_Cyp9f2
Dm_NP_523749.2_P450-6a8
Dm_NP_611002.3_Cyp6a20
Dm_NP_001286433.1_P450-6a17
Dm_NP_001286432.1_Cyp6a22
Dm_NP_001286199.1_Cyp6a14
Dm_NP_001285632.1_Cyp6a16
Dm_NP_001286145.1_Cyp6w1
Dm_NP_650327.1_Cyp6d5
Dm_NP_524598.1_P450-4c3
Dm_NP_525031.1_P450-4g1
Dm_NP_727531.2_Cyp4g15
Dm_NP_609129.2_Cyp4d21
Dm_NP_647723.2_Cyp4d20
Dm_NP_523961.1_P450-4d8
Dm_NP_001284806.1_P450-4d2
Dm_NP_726797.1_P450-4d1
Dm_NP_476907.2_P450-4d1
Dm_NP_573003.2_Cyp4s3
Dm_NP_524771.1_P450-4e1
Dm_NP_610473.1_Cyp4p3
Dm_NP_572780.3_Cyp311a1
MS_CYP63
MS_CYP70
MS_CYP34
MS_CYP67
MS_CYP35
Dm_NP_650783.2_Cyp12a4
Dm_NP_611370.2_Cyp12b2
Dm_NP_649151.1_Cyp305a1
Dm_NP_523403.2_P450-18a1
Dm_NP_731751.1_Cyp304a1
Dm_NP_001104460.2_spookier
MS_CYP62
MS_CYP61
MS_CYP60
MS_CYP11
MS_CYP65
MS_CYP66
MS_CYP64
MS_CYP1
MS_CYP16
MS_CYP17
MS_CYP15
MS_CYP91
MS_CYP90
Dm_NP_001260129.1_P450_reductase
0.4

Figure S5. Phylogenetic tree of 146 and 141 CYP proteins identified from MP and MS, respectively, with the 31 drosophila CYP proteins. Protein sequences were aligned using MAFFT. The phylogenetic tree was generated by IQ-TREE using maximum likelihood method, LG+I+G4 substitution model, and bootstrap with 1,000 iterations. The phylogenetic tree was visualized by the FigTree (version 1.4.4) progra

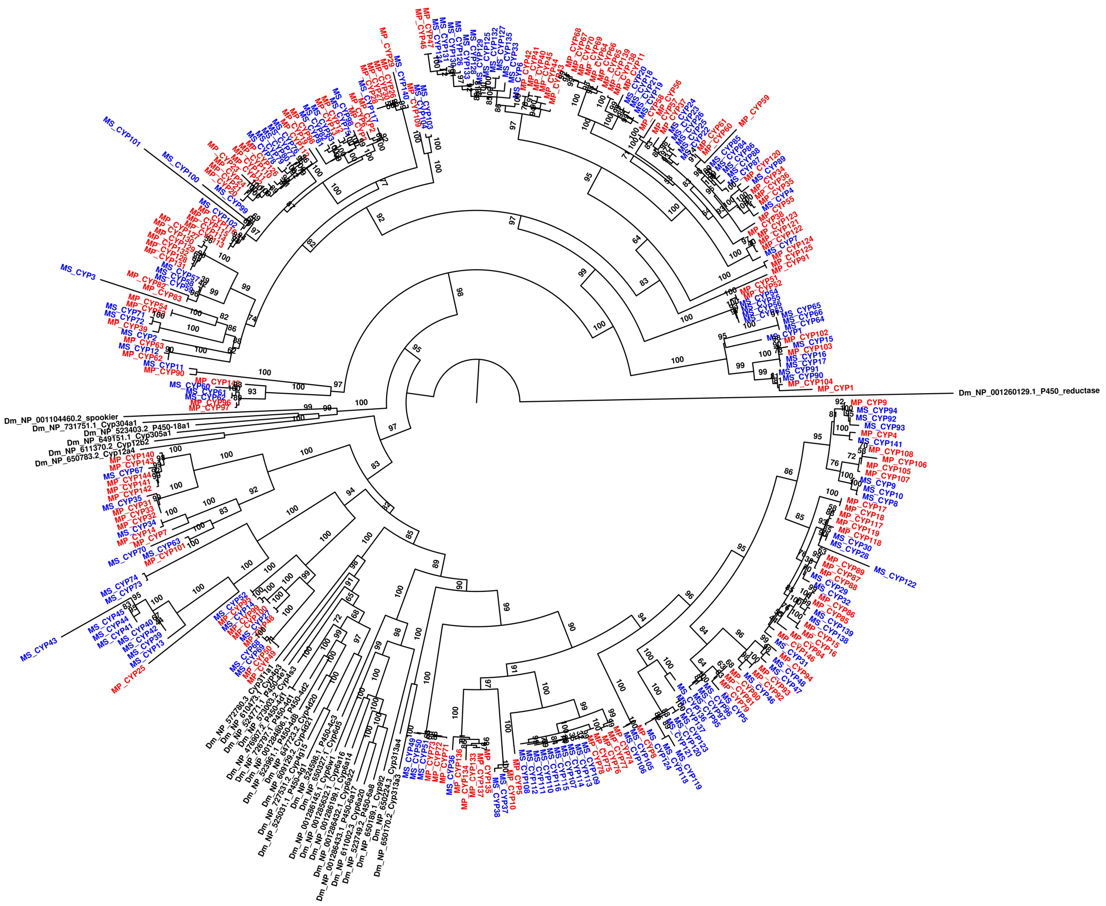

Supplement: Supplementary file 1 [file ijms-22-02085-s001.zip › SI-revise/ijms-1096217 SI-edit - Copy.pdf]
